# Transplantation of Eyes

**Published:** 1921-08-13

**Authors:** 


### Transplantation of Eyes.

A Hungarian student named Koppányi, working under Professor Przibram at the Biological Research Institute in Vienna, has recently been performing some rather sensational surgical operations. He has been transplanting eyes in salamanders and fishes and rats. Even in the case of the rat the operation has been of the simplest. He has merely removed a rat's eyes and placed the eyeballs of another rat in the empty sockets, putting a stitch through the eyelid to prevent the new eyeball from falling out. In twenty-four hours the optic nerve stumps effect a preliminary junction, and soon blood vessels grow into the eye and organic integrity seems entirely restored. Microscopic examination of eyes which have been grafted in this way show that the lens, retina, and all the tissues of the eye remain normal and intact.

Of course, the union of the optic nerve, the ingrowth of blood vessels, and the apparent histological integrity of the eye do not necessarily imply the restoration of the nerve tracts on which vision depends; but various facts suggest that a grafted eye is sensible to light and possesses to some extent the faculty of vision. The writer was shown one of the rats by Professor Przibram. The eyes were black and beady, with no obvious peculiarities save that there was slight proptosis of the left eye and that the pupil was rather excentric. The rat ran briskly about, leapt from the hand into the cage, and behaved in all respects like a rat with normal vision. Professor Przibram stated that a blind rat would never by any chance leap into the cage. But more conclusive even than the behaviour of the rat was the behaviour of its iris, for the light reflex could be clearly demonstrated. The success of this operation on a warm-blooded animal like the rat naturally opens up the question of the possibility of transplantation of the eye in human subjects. It may eventually be possible, but the transplantation of a human eye is a very different thing from the transplantation of the eye of a rat. The eye of a rat is embryonic, plastic, small; the human eye is a hundred times as large, and has lost all the developmental proclivities of an embryonic stage. The human eye has comparatively little independent vitality and comparatively small capacity for repair, and if placed in an empty socket would certainly degenerate and die before nervous and vascular union could be restored. Further, the human eye has multifarious neural connections with the brain, and even if the eye became sensitive to light and colour, it is extremely unlikely that the cerebral connections could be reconstituted. The man might be aware of light and colour and form, and yet be mentally blind. Still, Koppányi's experiments are only in their infancy and one cannot tell to what they may ultimately lead. Meantime they are at least a remarkable instance of the *vis medicatrix naturæ*.

